# Acupuncture Induces Divergent Alterations of Functional Connectivity within Conventional Frequency Bands: Evidence from MEG Recordings

**DOI:** 10.1371/journal.pone.0049250

**Published:** 2012-11-09

**Authors:** Youbo You, Lijun Bai, Ruwei Dai, Chongguang Zhong, Ting Xue, Hu Wang, Zhenyu Liu, Wenjuan Wei, Jie Tian

**Affiliations:** 1 Intelligent Medical Research Center, Institute of Automation, Chinese Academy of Sciences, Beijing, China; 2 Life Science Research Center, School of Electronic Engineering, Xidian University, Xi'an, Shaanxi, China; National Research & Technology Council, Argentina

## Abstract

As an ancient Chinese healing modality which has gained increasing popularity in modern society, acupuncture involves stimulation with fine needles inserted into acupoints. Both traditional literature and clinical data indicated that modulation effects largely depend on specific designated acupoints. However, scientific representations of acupoint specificity remain controversial. In the present study, considering the new findings on the sustained effects of acupuncture and its time-varied temporal characteristics, we employed an electrophysiological imaging modality namely magnetoencephalography with a temporal resolution on the order of milliseconds. Taken into account the differential band-limited signal modulations induced by acupuncture, we sought to explore whether or not stimulation at Stomach Meridian 36 (ST36) and a nearby non-meridian point (NAP) would evoke divergent functional connectivity alterations within delta, theta, alpha, beta and gamma bands. Whole-head scanning was performed on 28 healthy participants during an eyes-closed no-task condition both preceding and following acupuncture. Data analysis involved calculation of band-limited power (BLP) followed by pair-wise BLP correlations. Further averaging was conducted to obtain local and remote connectivity. Statistical analyses revealed the increased connection degree of the left temporal cortex within delta (0.5–4 Hz), beta (13–30 Hz) and gamma (30–48 Hz) bands following verum acupuncture. Moreover, we not only validated the closer linkage of the left temporal cortex with the prefrontal and frontal cortices, but further pinpointed that such patterns were more extensively distributed in the ST36 group in the delta and beta bands compared to the restriction only to the delta band for NAP. Psychophysical results for significant pain threshold elevation further confirmed the analgesic effect of acupuncture at ST36. In conclusion, our findings may provide a new perspective to lend support for the specificity of neural expression underlying acupuncture.

## Introduction

Acupuncture is one of the most important therapeutic modalities in Traditional Chinese Medicine (TCM), which treats patients by utilizing thin needles inserted into specific anatomical points named acupoints and then twirled manually [1]. Its treatments for postoperative and chemotherapy-induced nausea and vomiting and for postoperative dental pain are promising, and it can also be a beneficial adjunct or alternative treatment for drug addiction, stroke rehabilitation and chronic pain [2], [3]. One recent NIH survey in the USA demonstrated the sharply increased percentage of patients visiting acupuncturists from 27.2 per 1000 in 1997 to 79.2 per 1000 in 2007 [4]. In spite of its gaining popularity, however, it remains elusive on the scientific explanation about the neural mechanisms underlying the efficacy of acupuncture, hindering its profound significance in modern medical practice. To unveil the underlying biological mechanism would facilitate better acceptance and integration of this therapeutic modality into the practice of modern medicine.

One of the most highly attention-grabbing controversies focuses on acupoint specificity, which lies in the crucial position of traditional acupuncture theory. Based upon TCM, twirling needles at acupoints can correct imbalances in the flow of *qi* through channels known as meridians, while stimulation at points non-meridian points have little modulation effects [1]. In other words, the clinical effectiveness of acupuncture per se is said to depend on the specific placement of the needles [5]. However, scientific representation on acupoint specificity remains debatable in contemporary biomedical information [2], [6]. There are some pioneers, of whom Cho was one of the first, to find that the visual cortex could be activated by peripheral acupuncture at visually associated acupoints other than nearby non-meridian points [7], [8]. On the contrary, several recent studies illustrated no significant difference in functional Magnetic Resonance Imaging (fMRI) signal changes in acupuncture whether at vision-related or hearing-related acupoints compared with non-meridian points [9], [10]. Further work is therefore needed to elucidate the neurological basis of acupoint specificity so as to promote better acceptance of acupuncture as a viable clinical treatment.

During the last few decades, advances in non-invasive imaging techniques have significantly boosted neuroscience research, among which fMRI has been the dominant tool for exploring brain activity [7], [10], [11], [12], [13], [14]. Nevertheless, it is limited by its intrinsic nature of indirect assessments on cerebral metabolism and poor temporal resolution due to the protracted hemodynamic response [15]. Therefore, although previous fMRI studies have been of great assistance in spatially identifying function-associated brain regions [8], [9], [16], [17], [18], mechanisms underlying acupuncture may be unveiled incompletely considering its prolonged effects and time-varied temporal characteristic [12]. To introduce a more direct measurement of brain activity, as a result, may be of profound significance. Recently, it has been widely acknowledged that electroencephalography (EEG) and magnetoencephalography (MEG) enable us to monitor the dynamic neural activity of the whole brain, through which the electric/magnetic fields induced by the neuronal current flow in the brain are directly measured above the scalp [19], [20]. They both have a smaller time scale than fMRI on the order of milliseconds, presenting a much more refined perspective to track the transient neural activity [21]. Besides, since greater amounts of temporal information are being provided, it is more suitable to alternatively investigate from the concept of functional connectivity [22], [23], [24]. In particular, compared with EEG, MEG has more advantages to assess functional interactivity without distortion of magnetic fields by inhomogeneous conductivity or the need of a reference electrode [25].

The current study was developed to explore whether or not divergent alteration of functional connectivity exists following verum acupuncture (Stomach Meridian 36, ST36) relative to sham acupuncture (non-meridian point, NAP). Since previous electrophysiological investigations have demonstrated differential band-limited signal changes brought about by acupuncture [26], [27], [28], functional connectivity was sought in the present study with the hypothesis that distinct alteration patterns would be illustrated in response to the verum and sham acupuncture procedure within delta (0.5–4 Hz), theta (4–8 Hz), alpha (8–13 Hz), beta (13–30 Hz) and gamma bands (30–48 Hz).

## Materials and Methods

### Subjects

In order to reduce the inter-subject difference, 28 Chinese right-handed healthy college students (14 males, 14 females, aged 24.5±1.8 years) selected from a homogeneous group were enrolled in this study. They were all acupuncture naïve. None of them had a history of major medical illness, head trauma, neuropsychiatric disorders, nor did they use any prescription medications within the last month according to a questionnaire they filled out. All subjects gave written, informed consent after the experimental procedures had been fully explained. The research procedures were approved by the Tiantan Hospital Subcommittee on Human Studies and conducted in accordance with the Declaration of Helsinki.

### Experimental paradigm

Twenty-eight participants were evenly divided into two groups, being matched by age and gender. Every subject received only once acupuncture stimulation. They were instructed to sit comfortably in a dark and magnetically shielded room with their eyes closed and asked to remain relaxed without engaging in mental tasks.

**Figure 1:**
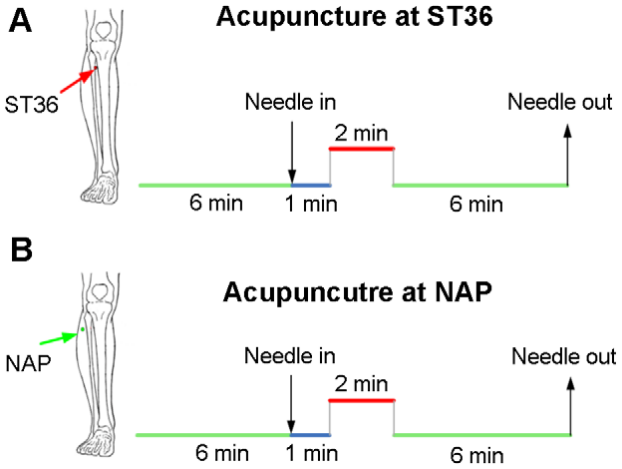
Experimental paradigm. Panel A indicates that acupuncture stimulation was performed at acupoint ST36 on the right leg (Zusanli, arrow pointing to the red dot). Panel B indicates that needling was performed at an adjacent nonacupoint on the right leg (NAP, arrow pointing to the green dot). The red line refers to needle administration, and the blue line represents no acupuncture manipulation but with needles inserted, while the green long line indicates a 6 min resting state or post-stimulus resting state. In this study, the two 6 min resting epochs were employed, while the rest were used for further analysis.

The experiment consisted of two functional runs. The resting-state run lasted 6 min. Acupuncture in both groups employed the single-block design paradigm, incorporating a 2 min needle manipulation, preceded by a 1 min rest epoch and followed by another 6 min resting scan (needle was kept in place without manipulation). See Fig. 1 for details. The two 6 min data scans were used in the present study. Acupuncture was performed at acupoint ST36 on the right leg, which has been proven to show great efficacy in pain-management in humans [29], [30]. It is located four finger breadths below the lower margin of the patella and one finger breadth laterally from the anterior crest of the tibia (arrow pointing to the red dot in Fig. 1). When acupuncture is executed, in classical literature, it is guided by the two classical manipulation procedures named “tonifying” and “reducing” [1]. The former is the reinforcing method of treatment which is performed by a comparatively weak stimulation to increase energy to the body, while the latter is the reducing method conducted by a comparatively strong stimulation to decrease energy. Clinically, the practice difference mainly lies in the fact that “tonifying” is the counter-clockwise rotation of needles while “reducing” is done clockwise [31]. In the present study, a balanced “tonifying and reducing” technique was utilized as twirling the needle clockwise and counter-clockwise equally [11]. Verum acupuncture was delivered using a sterile disposable 38 gauge stainless steel acupuncture needle, 0.2 mm in diameter and 40 mm in length, which was inserted perpendicularly into the skin surface at a depth of 1.5–2.5 cm. Sham acupuncture was exerted with needling at a nearby NAP (2–3 cm apart from ST36, arrow pointing to the green dot), with needle depth (1.5–2.5 cm), stimulation intensity (twirling needle for 2 min at the rate of 60 cycles/min) and manipulation procedure (balanced “tonifying and reducing” technique) all identical to those used in verum acupuncture. The whole procedure was performed by the same experienced and licensed acupuncturist on all participants.

According to TCM, the sensation induced by twirling needles at the acupoints is asserted as “*De-qi*”, which is essential to the efficacy of acupuncture [32]. As a concurrent psychophysical analysis, the MGH Acupuncture Sensation Scale (MASS) was utilized in the present study to quantify the subjective “*De*-*qi*” sensations, including throbbing, aching, soreness, heaviness, fullness, warmth, coolness, numbness, tingling, dull or sharp pain, pressure and one blank row for subjects to add their own observations if the above descriptors did not embody the sensations they experienced during the stimulation [33], [34]. The sensation rates ranged from 0 to 10 (0 = no sensation, 1–3 = mild, 4–6 = moderate, 7–8 = strong, 9 = severe and 10 = unbearable sensation). Spreading of any sensation was noted in a binary fashion and coded as follows: 1—spreading reported; 0—spreading not reported. As ST36 is a commonly used acupoint for pain control in clinical practice, we evaluated pain threshold changes as well during the experiment. Pain was induced by modified potassium iontophoresis with gradually increasing anodal currents, which has been accepted as a reliable measurement of pain tolerance [35], [36]. The iontophoretic pain generator mainly consists of a computer-controlled constant current source, with the ability to deliver a selected amount of current ranging from 0 to 5.0 mA. Intensity levels were graded in 0.2-mA steps [37]. The pain threshold was estimated by the current needed to produce pain [38]. Measurements for each subject were taken every 10 min for 60 min separately just before scanning and after the “*De*-*qi*” questionnaire was completed. The results of 6 scores in each condition were averaged respectively as each subject's pain threshold.

### MEG data acquisition

The MEG data were recorded while subjects were comfortably seated inside a magnetically shielded room using a 151-channel whole-head MEG system (CTF Systems Inc., Port Coquitlam, BC, Canada). Average distance between sensors in this system was 3.1 cm. The head position was monitored during the measurement using head position indicator coils. MEG data were recorded at the sample rate of 600 Hz. During the recording, participants were instructed to close their eyes to reduce artifact signals due to eye movements, but remained awake as much as possible. Subjects wore earplugs throughout the experiment to attenuate any sounds heard from outside of the MEG room. The investigator and MEG technician checked the signal on-line and observed the participants using a video monitor. At the beginning and end of each recording, the head position relative to the coordinate system of the helmet was recorded by leading small alternating currents through three head position coils attached to the left and right pre-auricular points and the nasion on the subject's head. If any subject's head moved more than 5 mm during the experiment, data from that subject would be discarded from further analysis. It turns out for all of the participants that the difference between the sensor locations evaluated during the whole experiment was not obvious, confirming a relatively stable head position.

### MEG data analysis

A third-order gradient noise reduction (computed with CTF software) was applied on line to the MEG signals. MEG data were then digitally filtered off-line with a band-pass of 0.5–48 Hz and further down sampled to 300 Hz. Subsequently, data were band-passed into the following frequency ranges: delta (0.5–4 Hz), theta (4–8 Hz), alpha (8–13 Hz), beta (13–30 Hz) and gamma bands (30–48 Hz) [39], [40]. The 6-min MEG scanning data of each subject before and after acupuncture were selected and split into 6 1-min long epochs respectively. For further off-line processing, the preprocessed MEG data were converted to ASCII files. The following processing was executed for the 6 epochs separately and results were averaged for each subject.

The MEG channels were grouped into 10 regions of interest (ROIs) roughly corresponding to the major cortical areas (frontal, temporal, central, parietal and occipital for each hemisphere). Two of the original 151 channels were not available due to technical problems. Besides, the 9 midline channels were left out of clustering, leaving a total of 140 channels divided over 10 ROIs for further analysis. The band-limited power (BLP) of each channel, defined as the envelope of the band-limited signal, was calculated by first applying the Hilbert transform to the band-limited signal and then taking the absolute value of the resultant complex helical sequence [41]. The BLP signals were thereafter low-pass filtered (cutoff = 8 Hz) to eliminate ringing [42]. With EEG/MEG data, the Hilbert transform has been adopted to estimate power and phase in narrow frequency bands [43], [44]. It is a convolution of the data with the kernel 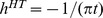, which is equivalent to altering all phases of the original signal components by 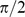. The analytic signal is a complex function given by 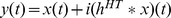, where 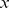 is the original EEG/MEG data of one channel or voxel/equivalent dipole for reconstructed sources and 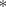 is the convolution operator [45].

For each frequency band of interest, the pair-wise temporal correlations between the BLP signals were computed using Pearson's correlation. The end result is a 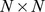 matrix with 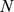 equals to 140, where each entry 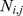 contains the correlation value for the channels 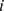 and 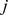. Results of the 6 epochs were averaged to obtain the mean correlation matrix for each subject both preceding and following acupuncture. Group analysis was carried out by one-sample *t*-test with the null hypothesis set as there is no significant functional connectivity within or between brain regions. The same items from individual connectivity matrix (with same row index and column index) were taken together for one-sample *t*-test. After obtaining the *P*-value for each *t*-test, false discovery rate (FDR) criterion was then deployed to make the correction for multiple comparisons. The FDR concept was formally described by Benjamini to control the expect proportion of incorrectly rejected null hypotheses [46], [47]. It was conducted as follows. Given that there are *m* hypotheses to be tested 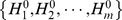, 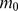 are true null hypotheses, the number and identity of which are unknown. The other 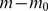 hypotheses are false. Denote the corresponding random vector of test statistics 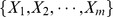, and the corresponding *P*-values 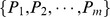. It was shown that the following procedure controls the FDR at level *q*: 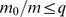 (*q* was set as 0.05 in our study). Let 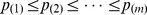 be the ordered observed *P*-values. Define 
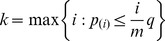
, and reject 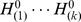 [48]. After FDR procedure, further averaging was carried out to evaluate long distance intra- and inter-hemispheric and short distance local measures both preceding and following acupuncture. The short distance interactivity was computed as the average correlation coefficient between all sensor pairs within one region, while long distance connectivity (8 intrahemispheric: fronto-temporal, fronto-parietal, parieto-occipital and occipito-temporal; 5 interhemispheric: central, frontal, occipital, parietal and temporal) was obtained from sensor pairs where one sensor was in one region, and the other was in another [49], [50], [51]. Finally, significant alterations induced by acupuncture for either ST36 or NAP group was evaluated for each band by means of a paired *t*-test with threshold at *P*<0.05 in SPSS 17.0 software package for Windows.

## Results

### Psychophysical responses

**Figure 2:**
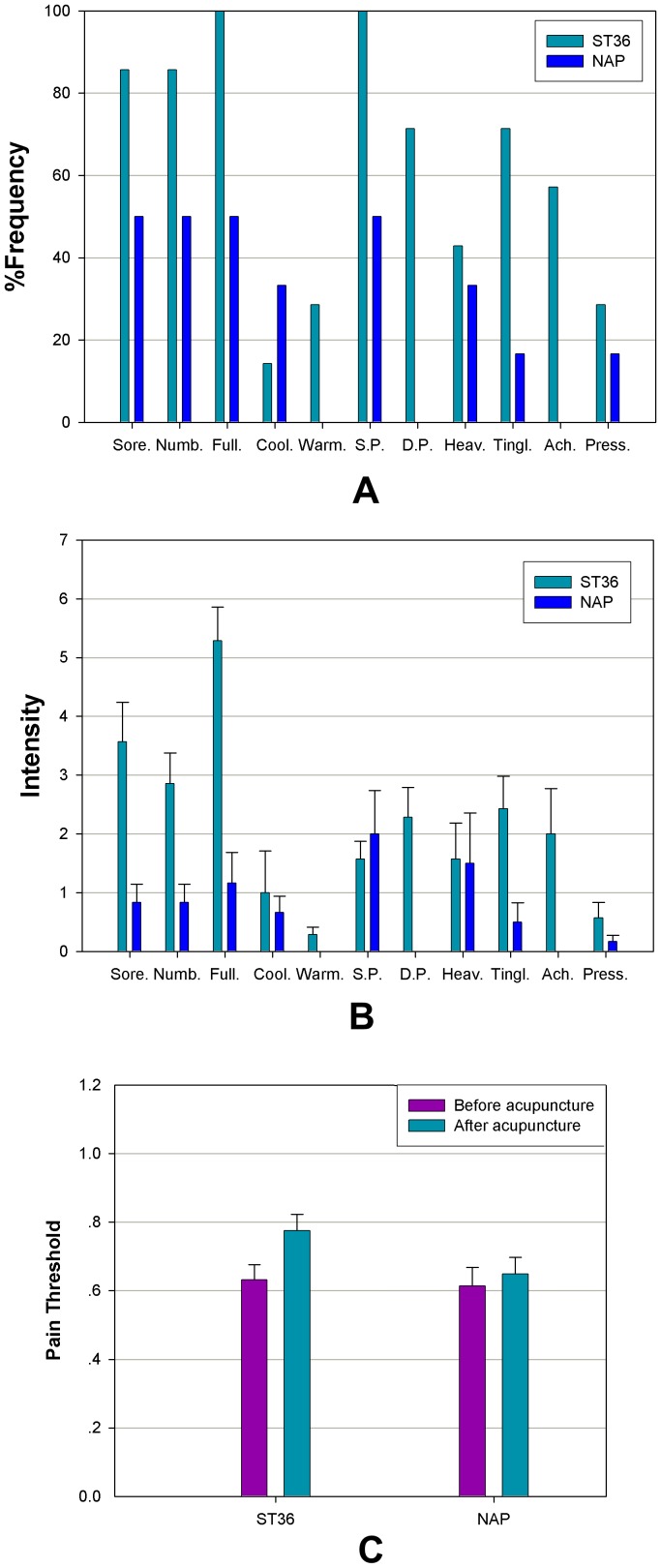
Averaged psychophysical response. A. The percentage of subjects that reported the given sensations. The frequency of aching was found to be greater following acupuncture at ST36. B. The intensity of sensations measured by average score (with standard error bars) on a scale from 0 denoting no sensation to 10 denoting an unbearable sensation. Sore, soreness; Numb, numbness; Full, fullness; Cool, coolness; Warm, warmth; SP, sharp pain; DP, dull pain; Heav, heaviness; Tinl, tingling; Ach, aching; Press, pressure. C. The pain threshold evaluated by average score (with standard error bars) before and after acupuncture at ST36 and NAP. Significant elevation of the pain threshold was observed following acupuncture at ST36.

The prevalence of subjective “*De-qi*” sensations was expressed as the percentage of individuals in the group who reported the given sensations (Fig. 2A). The intensity was expressed as the average score±standard error (Fig. 2B). No subject opted to add an additional descriptor in the blank row provided. The occurrence frequency of all sensations except coolness was found to be greater during verum acupuncture relative to the sham group. The overall stimulus intensities (mean±SE) were greater for ST36, exhibiting a stronger “*De-qi*” sensation in verum acupuncture. The pain threshold measured for each group per condition was also denoted as the mean±SE. By comparing the pain threshold evaluated preceding and following acupuncture within each group, we illustrated the significant elevation of the pain threshold for acupuncture at ST36 (*P* = 0.023, paired *t*-test), while no conspicuous changes were identified for NAP following acupuncture (*P* = 0.620, paired *t*-test).

### Alteration of functional connectivity

For each condition preceding or following acupuncture, the temporal correlations of band-limited power (BLP) signals were first computed for every pair of MEG channels in each frequency band and then grouped into local and long-distance couplings. The grand averaged local and long-distance couplings for the two conditions in each group were taken in for further statistical analysis.

**Figure 3:**
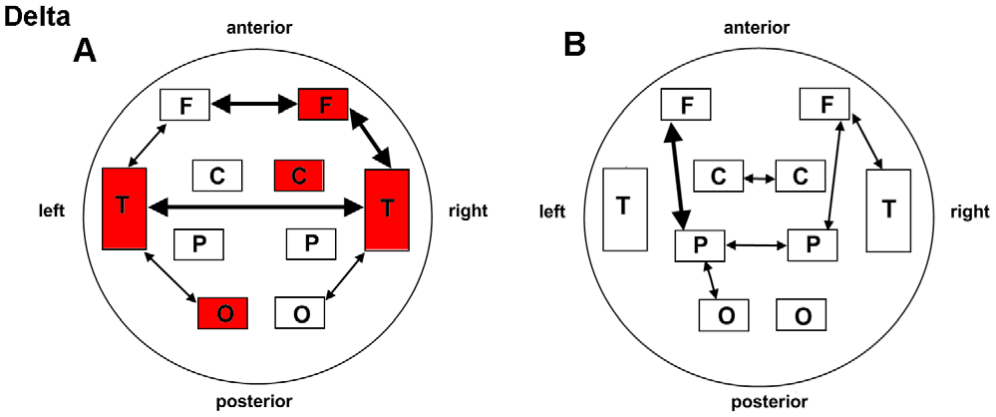
Schematic illustration of BLP correlation alterations for the delta band. A. ST36 group. B. NAP group. Lines correspond to significant changes for the average Band-Limited Power (BLP) correlation induced by acupuncture and squares to significant change in the local BLP correlation (red: local increase in the BLP correlation following acupuncture; thin line: *P*<0.05; thick line: *P*<0.01; significance is based upon a paired *t*-test).

Among the 5 frequency bands either for verum or sham acupuncture, our results demonstrated dominant enhanced connectivity within the delta band (0.5–4 Hz). As illustrated in Fig. 3 and Table 1, local potentiation of BLP correlations following acupuncture at ST36 was identified over the right frontal (*P* = 0.005), right central (*P* = 0.009), and left occipital (*P* = 0.001) areas, as well as the left (*P* = 0.0001) and right (*P* = 0.007) temporal regions, none of which could be detected in the NAP group. Although statistical analyses in both groups presented tighter linkage of the right frontal and temporal regions after stimulation (ST36: *P* = 0.008; NAP: *P* = 0.033), distinct changes in long distance connections were revealed as well. To be specific, left fronto-temporal connectivity was enhanced only for ST36 (*P* = 0.019). Elevated connectivity was detected following stimulation at ST36 of the bilateral frontal (*P* = 0.005) and temporal (*P* = 0.007) regions and the occipito-temporal linkage in both hemispheres (left: *P* = 0.014; right: *P* = 0.020), while an increased connection was indicated alone in the NAP group between the bilateral central (*P* = 0.043) and parietal (*P* = 0.017) regions as well as the left parieto-occipital connections (*P* = 0.048).

**Table 1:** Functional connectivity within and between main regions and statistical results in the delta bands for the ST36 and NAP groups.

	Delta							
	Group ST36				Group NAP			
Areas	B_rest	P_rest	*t* value	*P* value	B_rest	P_rest	*t* value	*P* value
LC	0.3902±0.0525	0.4215±0.0612	1.949	0.073	0.4247±0.0864	0.4579±0.0937	1.693	0.114
LF	0.5480±0.1057	0.5908±0.0958	1.961	0.072	0.4953±0.1029	0.5077±0.1127	0.613	0.551
LO	**0.3930±0.0482**	**0.4671±0.0748**	**4.579**	**0.001**	0.4631±0.1203	0.4979±0.1018	1.424	0.178
LP	0.5383±0.0670	0.5721±0.0692	2.063	0.060	0.5481±0.0926	0.5777±0.0996	2.130	0.053
LT	**0.3650±0.0840**	**0.4387±0.0915**	**5.416**	**0.0001**	0.3999±0.0855	0.4204±0.1057	0.904	0.383
RC	**0.3760±0.0801**	**0.4191±0.0730**	**3.055**	**0.009**	0.4287±0.1261	0.4727±0.1038	1.947	0.073
RF	**0.4830±0.1551**	**0.5917±0.1316**	**4.761**	**0.0004**	0.5248±0.1133	0.5546±0.1545	1.088	0.297
RO	0.3940±0.0684	0.4176±0.0475	1.238	0.238	0.4862±0.1740	0.4738±0.4738	−0.539	0.599
RP	0.4592±0.0580	0.4670±0.0869	0.483	0.637	0.4986±0.1353	0.4962±0.1146	−0.341	0.868
RT	**0.3671±0.1044**	**0.4361±0.1276**	**3.063**	**0.009**	0.4110±0.1107	0.4461±0.1150	1.689	0.115
LF_LP	0.1789±0.0533	0.1767±0.0914	−0.119	0.907	**0.1595±0.0910**	**0.2096±0.0645**	**3.131**	**0.008**
LF_LT	**0.2399±0.0568**	**0.2853±0.0521**	**2.670**	**0.019**	0.2373±0.0733	0.2541±0.0757	0.804	0.436
LO_LP	0.2020±0.0617	0.2363±0.0871	1.693	0.114	**0.2528±0.1492**	**0.2926±0.1146**	**2.187**	**0.048**
LO_LT	**0.1451±0.0335**	**0.1965±0.0663**	**2.840**	**0.014**	0.1904±0.0914	0.2037±0.0457	0.562	0.583
RF_RP	0.1428±0.0422	0.1755±0.0995	1.371	0.194	**0.1751±0.1063**	**0.2157±0.0799**	**2.561**	**0.024**
RF_RT	**0.2314±0.0820**	**0.3007±0.1019**	**3.155**	**0.008**	**0.2751±0.0860**	**0.3262±0.1027**	**2.382**	**0.033**
RO_RP	0.1730±0.0445	0.2027±0.0516	1.812	0.093	0.2458±0.1913	0.2522±0.1559	0.301	0.768
RO_RT	**0.1609±0.0445**	**0.2164±0.0595**	**2.653**	**0.020**	0.2049±0.0863	0.2161±0.0593	0.433	0.672
LC_RC	0.1626±0.0428	0.1775±0.0616	0.903	0.383	**0.1789±0.1003**	**0.2322±0.0709**	**2.239**	**0.043**
LF_RF	**0.3104±0.1285**	**0.3755±0.1183**	**3.369**	**0.005**	0.2740±0.0890	0.3067±0.1145	1.169	0.263
LO_RO	0.1637±0.0508	0.1892±0.0763	1.149	0.271	0.2314±0.1829	0.2209±0.1128	−0.436	0.670
LP_RP	0.1516±0.0567	0.1525±0.0748	0.056	0.956	**0.1677±0.1118**	**0.2200±0.0976**	**2.724**	**0.017**
LT_RT	**0.1811±0.0572**	**0.2349±0.0740**	**3.163**	**0.007**	0.2552±0.0961	0.2791±0.1143	1.083	0.298

Significant differences are indicated in bold (*P*<0.05). L = left, R = right. C = central, F = frontal, O = occipital, P = parietal, T = temporal. B_rest, resting data before acupuncture. P_rest, resting data after acupuncture.

**Figure 4:**
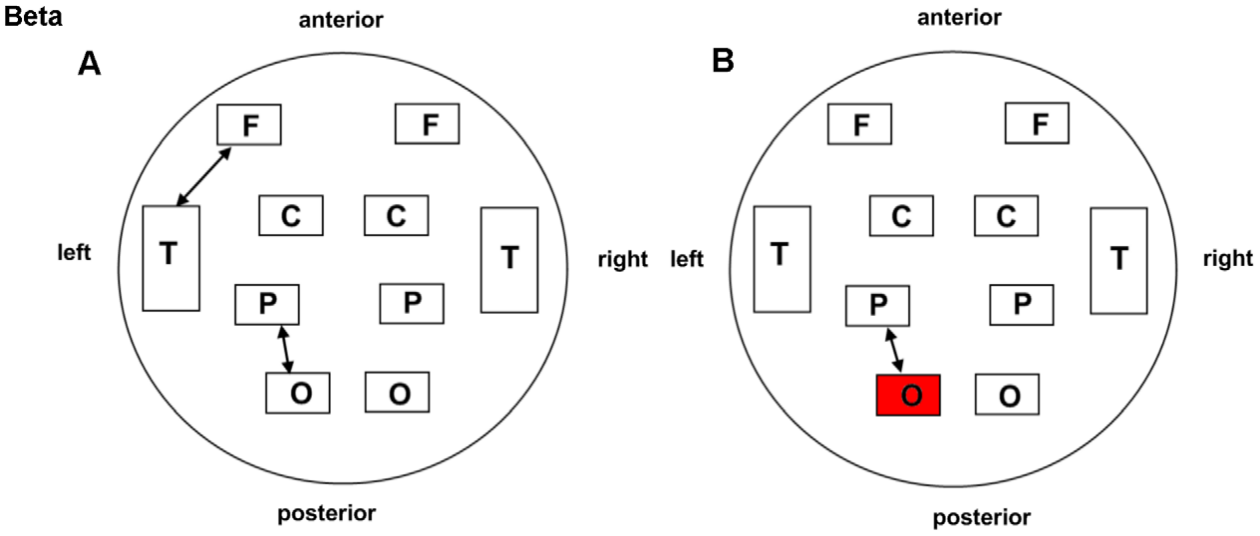
Schematic illustration of BLP correlation alterations for the beta band. A. ST36 group. B. NAP group. Lines correspond to significant changes for the average Band-Limited Power (BLP) correlation induced by acupuncture and squares to significant change in the local BLP correlation (red: local increase in the BLP correlation following acupuncture; thin line: *P*<0.05; thick line: *P*<0.01; significance is based upon a paired *t*-test).

Regarding the beta band (13–30 Hz), both groups displayed a prominently increased left parieto-occipital connection (*P* = 0.035 for ST36; *P* = 0.031 for NAP). The left fronto-temporal connections for ST36 (*P* = 0.022) and local connectivity in the left occipital region for NAP (*P* = 0.048) were found to be enhanced following acupuncture. These interaction effects are illustrated schematically in Fig. 4 and Table 2.

**Table 2:** Functional connectivity within and between main regions and statistical results in the beta bands for the ST36 and NAP groups.

	Beta							
	Group ST36				Group NAP			
Areas	B_rest	P_rest	*t* value	*P* value	B_rest	P_rest	*t* value	*P* value
LC	0.4420±0.0750	0.4431±0.0748	0.074	0.942	0.4373±0.0690	0.4328±0.0872	−0.540	0.598
LF	0.4104±0.0991	0.4186±0.0860	1.381	0.190	0.3898±0.0786	0.3875±0.0811	−0.275	0.788
LO	0.4337±0.0490	0.4480±0.0548	1.658	0.121	**0.4188±0.0631**	**0.4335±0.0778**	**2.183**	**0.048**
LP	0.6715±0.0574	0.6823±0.0673	0.880	0.395	0.6398±0.0604	0.6414±0.0724	0.250	0.806
LT	0.3608±0.0470	0.3586±0.0511	−0.402	0.694	0.3554±0.0674	0.3560±0.0752	0.089	0.931
RC	0.4231±0.0791	0.4164±0.0955	−0.412	0.687	0.4118±0.0675	0.4043±0.0874	−0.677	0.510
RF	0.4822±0.0680	0.4793±0.0670	−0.284	0.781	0.4545±0.0808	0.4492±0.0821	−0.692	0.501
RO	0.4175±0.0582	0.4299±0.0717	1.152	0.270	0.4333±0.0564	0.4456±0.0615	1.609	0.132
RP	0.6959±0.0568	0.6987±0.0479	0.263	0.797	0.6779±0.0503	0.6788±0.0594	0.116	0.909
RT	0.3662±0.0324	0.3645±0.0278	−0.300	0.769	0.3600±0.0638	0.3511±0.0663	−1.303	0.215
LF_LP	0.0865±0.0389	0.1029±0.0505	1.879	0.083	0.0746±0.0371	0.0837±0.0452	1.791	0.097
LF_LT	**0.1382±0.0329**	**0.1537±0.0441**	**2.590**	**0.022**	0.1224±0.0461	0.1308±0.0411	1.803	0.095
LO_LP	**0.2126±0.0565**	**0.2354±0.0652**	**2.358**	**0.035**	**0.1810±0.0579**	**0.2011±0.0702**	**2.412**	**0.031**
LO_LT	0.1598±0.0388	0.1688±0.0474	1.272	0.226	0.1706±0.0617	0.1808±0.0762	1.712	0.111
RF_RP	0.0901±0.0338	0.1088±0.0474	1.834	0.090	0.0842±0.0447	0.0877±0.0473	0.585	0.568
RF_RT	0.1999±0.0407	0.199±0.0424	−0.001	0.999	0.1717±0.0663	0.1714±0.0616	−0.047	0.963
RO_RP	0.2176±0.0527	0.2373±0.0594	2.128	0.053	0.2061±0.0550	0.2182±0.0554	1.671	0.119
RO_RT	0.1539±0.0464	0.1631±0.0453	1.084	0.298	0.1747±0.0519	0.1739±0.0577	−0.089	0.930
LC_RC	0.1361±0.0495	0.1449±0.0605	0.972	0.349	0.1174±0.0435	0.1234±0.0572	0.838	0.417
LF_RF	0.1729±0.0455	0.1865±0.0561	1.791	0.097	0.1460±0.0582	0.1495±0.0572	0.597	0.561
LO_RO	0.1838±0.0530	0.1921±0.0670	0.792	0.443	0.1982±0.0776	0.2124±0.0941	1.614	0.130
LP_RP	0.2224±0.0562	0.2531±0.0755	1.693	0.114	0.2054±0.0504	0.2201±0.0492	1.640	0.125
LT_RT	0.1915±0.0500	0.1837±0.0532	−0.805	0.435	0.1904±0.0539	0.1881±0.0695	−0.303	0.767

Significant differences are indicated in bold (*P*<0.05). L = left, R = right. C = central, F = frontal, O = occipital, P = parietal, T = temporal. B_rest, resting data before acupuncture. P_rest, resting data after acupuncture.

**Figure 5:**
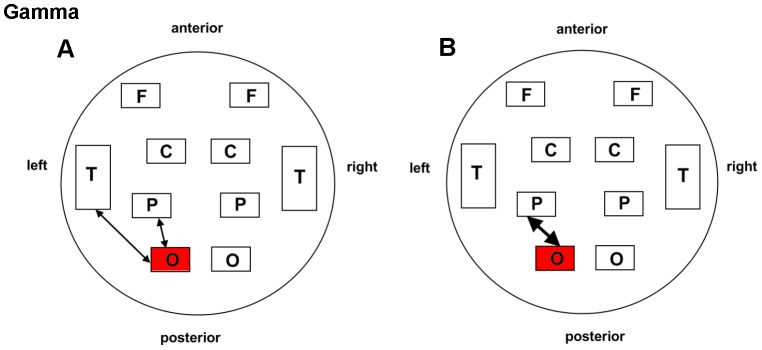
Schematic illustration of BLP correlation alterations for the gamma band. A. ST36 group. B. NAP group. Lines correspond to significant changes for the average Band-Limited Power (BLP) correlation induced by acupuncture and squares to significant change in the local BLP correlation (red: local increase in the BLP correlation following acupuncture; thin line: *P*<0.05; thick line: *P*<0.01; significance is based upon a paired *t*-test).

As for the gamma band (30–48 Hz), shared patterns of long and short distance interactivity alteration could be detected in both groups to certain extent (Fig. 5 and Table 3). Enhanced connection in the left parieto-occipital (ST36: *P* = 0.029; NAP: *P* = 0.008) and occipital regions (ST36: *P* = 0.034; NAP: *P* = 0.048) were illustrated for the two groups. Furthermore, an additional intrahemispheric connection of the left occipito-temporal region was increased following acupuncture in the ST36 group (*P* = 0.031) compared with NAP (*P* = 0.255).

**Table 3:** Functional connectivity within and between main regions and statistical results in the gamma bands for the ST36 and NAP groups.

	Gamma							
	Group ST36				Group NAP			
Areas	B_rest	P_rest	*t* value	*P* value	B_rest	P_rest	*t* value	*P* value
LC	0.2669±0.0856	0.2473±0.0798	−1.553	0.144	0.2777±0.0880	0.2656±0.0902	−1.584	0.137
LF	0.2187±0.0825	0.2104±0.0660	−0.884	0.393	0.2279±0.0934	0.2244±0.0892	−0.605	0.555
LO	**0.3412±0.0549**	**0.3658±0.0590**	**2.369**	**0.034**	**0.3163±0.0342**	**0.3256±0.0363**	**2.763**	**0.016**
LP	0.4430±0.0723	0.4307±0.0766	−0.886	0.392	0.4486±0.0879	0.4432±0.0883	−0.574	0.576
LT	0.2145±0.0333	0.2232±0.0321	1.850	0.087	0.2131±0.0483	0.2133±0.0454	0.068	0.947
RC	0.2281±0.0739	0.2112±0.0791	−1.672	0.118	0.2396±0.0753	0.2275±0.0789	−1.550	0.145
RF	0.2568±0.0768	0.2578±0.0762	0.109	0.915	0.2640±0.0904	0.2632±0.0797	−0.112	0.913
RO	0.3197±0.0410	0.3144±0.0309	−0.547	0.594	0.3127±0.0388	0.3151±0.0415	0.768	0.456
RP	0.4719±0.0726	0.4598±0.0813	−0.759	0.462	0.4822±0.0734	0.4696±0.0759	−1.279	0.223
RT	0.2260±0.0264	0.2309±0.0297	0.869	0.401	0.2212±0.0428	0.2213±0.0372	0.028	0.978
LF_LP	0.0297±0.0187	0.0331±0.0188	1.319	0.210	0.0338±0.0405	0.0368±0.0368	0.964	0.353
LF_LT	0.0489±0.0209	0.0546±0.0228	1.093	0.079	0.0528±0.0453	0.0558±0.0400	1.086	0.297
LO_LP	**0.1055±0.0176**	**0.1209±0.0266**	**2.460**	**0.029**	**0.0987±0.0332**	**0.1085±0.0317**	**3.099**	**0.008**
LO_LT	**0.0790±0.0198**	**0.0945±0.0255**	**2.411**	**0.031**	0.0768±0.0280	0.0816±0.0257	1.192	0.255
RF_RP	0.0325±0.0160	0.0353±0.0190	0.806	0.435	0.0327±0.0210	0.0315±0.0153	−0.423	0.679
RF_RT	0.0630±0.0219	0.0710±0.0247	1.894	0.081	0.0634±0.0373	0.0660±0.0261	0.452	0.659
RO_RP	0.1098±0.0214	0.1115±0.0212	0.449	0.661	0.1106±0.0280	0.1085±0.0296	−1.169	0.263
RO_RT	0.0734±0.0183	0.0780±0.0184	1.059	0.309	0.0692±0.0209	0.0714±0.0212	1.206	0.249
LC_RC	0.0534±0.0289	0.0488±0.0320	−1.261	0.229	0.0519±0.0317	0.0506±0.0341	−0.327	0.749
LF_RF	0.0639±0.0396	0.0679±0.0386	0.858	0.407	0.0689±0.0695	0.0689±0.0602	−0.002	0.999
LO_RO	0.1084±0.0352	0.1098±0.0330	0.143	0.889	0.0976±0.0381	0.1046±0.0405	1.865	0.085
LP_RP	0.1192±0.0302	0.1210±0.0326	0.319	0.755	0.1176±0.0449	0.1176±0.0393	0.008	0.994
LT_RT	0.0650±0.0245	0.0700±0.0269	1.189	0.256	0.0648±0.0374	0.0634±0.0346	−0.333	0.744

Significant differences are indicated in bold (*P*<0.05). L = left, R = right. C = central, F = frontal, O = occipital, P = parietal, T = temporal. B_rest, resting data before acupuncture. P_rest, resting data after acupuncture.

Additionally, both theta (4–8 Hz) and alpha (8–13 Hz) bands missed significant interaction alterations of functional connectivity in the ST36 and NAP groups (*P*>0.05). The overall findings indicated that acupuncture at different designated places may evoke differential alterations of functional connectivity within specific frequency bands.

## Discussion

It is noteworthy that when using MEG technology, we should always take into account the question whether the correlation measured between signals at different sensors can be interpreted with physiological interactions between different brain areas. This is the well-known problem of volume conduction effects [52], [53]. In other words, nearby MEG sensors have a high probability of capturing activity from common sources, and therefore may show spurious correlation. One possible solution is to estimate correlations between signals from restructured sources rather than from actual recorded signals. Nevertheless, there is to date no reliable way to choose the proper model to unambiguously solve the inverse problem [42], [54]. Apart from this, another approach is the adoption of measures of correlation that are not sensitive to volume conduction [55]. However, even this approach may not always be effective [56]. In the present study, we employed a pragmatic approach which has been generally adopted in resting-state MEG investigations, analyzing functional connectivity in sensor space and then grouping the sensor pairs in local and long-distance couplings [49], [50], [51], [53], [57]. Although precise correspondence with anatomical localization is to some extent limited, underlying cortical areas are to be considered as indicative since the ROIs are based upon the very extra-cranial position of the MEG sensors [39], [50].

Acupuncture-induced modulations on functional connectivity have already been illustrated in previous fMRI investigations [58], [59], [60]. Given that fMRI is naturally an indirect imaging tool, we attempted to seek whether or not such alterations would be directly observed using an electrophysiological imaging modality, among which MEG being the most suitable for estimating functional connectivity [40], [61], [62]. The present MEG study was conducted with the objective of exploring the global differences in the interregional functional connectivity induced by acupuncture within delta, theta, alpha, beta and gamma bands.

Although significant alterations for both the verum and sham groups were mainly confined to delta, beta and gamma bands, the functional connectivity within each presented distinct change patterns. One intriguing finding here is the increased degree of connectivity recorded by sensors overlying the left temporal cortex within the delta, theta and gamma bands. Compared to recent fMRI studies in which the temporal gyrus as well as the underlying amygdala and hippocampus were indicated as network hubs following verum acupuncture, with the advantage of MEG we observed that such modulation effects existed specifically within the above-mentioned three bands, among which delta was the most dominant [58], [59]. Another thought-provoking result is that in addition to previous investigations which illustrated enhanced interactions of the temporal gyrus with the frontal gyrus and prefrontal cortex following acupuncture either at ST36 or NAP, we further pinpointed that such an effect occurred only in the delta band for sham acupuncture, compared with the additional modulation effect in the beta band of the verum group [60]. Note that both groups presented somewhat shared alteration patterns for beta and gamma bands, mainly comprising of the parietal and occipital regions. This may further implicate the modulation of the resting state network by sham acupuncture [60]. Besides, it is speculated that the shared enhanced couplings may partly support the clinical experience that acupuncture at non-meridian points can also provide partial analgesia in chronic pain [63].

### Limitations

To the best of our knowledge, this MEG study is the first to demonstrate the global differences in functional connectivity alterations induced by acupuncture. However, due to the inverse problem currently not to be solved properly, this preliminary research did not involve the source reconstruction. Therefore, we are currently not able to exactly evaluate the anatomical correspondence to the temporal structures, which must be considered as a pitfall. As far as we know, there have been several MEG studies using this methodology which successfully illustrated differential functional connectivity patterns in pathological patients compared with normal control [49], [50], [51], [53]. As a result, although the analysis was conducted at the sensor space, our results to some extent can make a contribution to improving the knowledge about the functional specificity of acupuncture. In the future, to solve the inverse problem will be one of the main research interests so that more specific anatomical information would be dug out by source reconstruction and make further efforts to unveil the neurophysiological mechanism underlying acupuncture.
